# Ferroptosis in liver diseases: molecular mechanisms, biomarker potential, and clinical translation

**DOI:** 10.3389/fcell.2026.1865349

**Published:** 2026-07-22

**Authors:** Heng Tian, Yu Yang, Li Chen, Ran Wei, Wang Liu, Yuhan Liu, Jiaju Wang, Enba Zhuo, Kangsheng Gu, Jianwei Xing

**Affiliations:** 1 Department of Oncology, The First Affiliated Hospital of Anhui Medical University, Hefei, Anhui, China; 2 Department of Oncology of Integrated Traditional Chinese and Western Medicine, The First Affiliated Hospital of Anhui Medical University, Hefei, Anhui, China; 3 First Clinical Medical College, Anhui Medical University, Hefei, Anhui, China; 4 Department of General Surgery, Sanya Central Hospital (The Third People’s Hospital of Hainan Province), Sanya, Hainan, China; 5 Department of Oncology, Affiliated Drum Tower Hospital, Medical School of Nanjing University, Nanjing, Jiangsu, China; 6 Department of Anesthesiology, The Affiliated Hospital of Yangzhou University, Yangzhou University, Yangzhou, Jiangsu, China

**Keywords:** biomarkers, clinical translation, ferroptosis, liver diseases, precision medicine

## Abstract

Ferroptosis is a type of intracellular, iron-dependent cell death that differs from apoptosis, necrosis, and autophagy. Ferroptosis is characterized by excessive lipid peroxidation. Iron-dependent, non-apoptotic cell death is linked to several liver diseases. Although numerous ferroptosis-associated genes and pathways have been linked to liver disorders, the exact mechanisms by which ferroptosis contributes to disease initiation and progression remain incompletely understood. In this review, we discuss the initiation and role of ferroptosis in the pathophysiology of liver diseases, including acute liver injury, liver fibrosis, hepatocellular carcinoma, viral hepatitis, autoimmune hepatitis, alcohol-associated liver disease, and NAFLD/MASLD. We first describe the regulatory function of ferroptosis before highlighting its relevance and underlying processes in several distinct liver disorders. In addition, we briefly discuss the potential clinical relevance of ferroptosis-related molecules and pathways as candidate biomarkers and therapeutic targets in liver diseases, with emphasis on their possible value in biomarker discovery, patient stratification, disease evaluation, and clinical translation. We also emphasize the context-dependent role of ferroptosis, in which its induction may be therapeutically beneficial in hepatocellular carcinoma or activated hepatic stellate cells, whereas excessive hepatocyte ferroptosis may aggravate non-malignant liver injury.

## 1 Introduction

Liver disease is a leading cause of illness and mortality worldwide. The liver has remarkable regenerative capacity (Neshat et al., 2021). However, some illnesses overstimulate this regenerative capacity, leading to excessive accumulation of collagen and cellular matrix (Neshat et al., 2021). Approximately 300 million individuals in China suffer from liver illnesses, mainly viral hepatitis (mostly caused by the hepatitis B virus, HBV), nonalcoholic fatty liver disease (NAFLD), and alcohol-associated liver disease (ALD). Furthermore, the incidence of alcoholic and nonalcoholic fatty liver diseases has markedly increased (Wang et al., 2014). The second most common cancer in China is liver cancer. China accounts for 51% of all liver cancer fatalities worldwide; >383,000 deaths are reported annually (Wang et al., 2014). Ferroptosis is a form of iron-dependent regulated cell death that is closely associated with dysregulated metabolic pathways and disrupted iron homeostasis in liver diseases (Chen et al., 2022).

The liver is the metabolic center of the body; it supplies nutrients such as amino acids and lipids. An imbalance in these nutrients leads to oxidative stress and other complications, including cell death (Steinberg et al., 2025; Rao et al., 2023). Ferroptosis involves numerous metabolic processes in the liver (Chen et al., 2022; Zhou et al., 2022). For example, NADPH levels decrease significantly during ferroptosis (Nagakannan et al., 2025; Liu N. et al., 2024). The metabolic NADPH pathway prevents ferroptosis by facilitating GSH and CoQ10 production (Bersuker et al., 2019). Thus, NADPH may serve as a metabolic indicator associated with ferroptosis susceptibility (Chen et al., 2022; Mao and Gan, 2022).

Systemic iron homeostasis is strictly controlled to ensure sufficient dietary absorption of iron and avoid its harmful build-up (Galy et al., 2024; Frazer et al., 2025). Iron homeostasis is primarily regulated by hepcidin, which is secreted by the liver, the principal regulator of iron metabolism (Lakhal-Littleton and Peyssonnaux, 2025; Nemeth et al., 2004; Fisher et al., 2025).

Ferroptosis, a non-apoptotic iron-dependent programmed cell death, alters lipid peroxidation and iron metabolism. Cells that undergo ferroptosis do not exhibit the morphological characteristics of necrosis or apoptosis (Wu et al., 2023). Instead, they exhibit specific morphological characteristics such as small malformed mitochondria, crest reduction, membrane condensation, and outer membrane rupture (Lei et al., 2021).

Decreased antioxidative defense and elevated oxidative stress are the biochemical hallmarks of ferroptosis (Sun et al., 2023). Ferroptosis preserves cellular integrity more effectively than apoptosis. Therefore, it is used to trigger cell death at certain time periods. Ferroptosis-based cancer therapies that primarily target apoptotic pathways have shown significant promise in treating patients with cancer who have not responded to conventional treatments (Lei et al., 2021). Hepatic SLC39A14 accelerated iron-induced ferroptosis by trafficking NTBI in the liver of hepatic Trf-mutant mice. This revealed a novel molecular mechanism underlying the pathogenesis of liver fibrosis and cirrhosis (Chen et al., 2022).

Ferroptosis plays a role in several pathophysiological conditions, including cancer, dementia, cardiovascular disease, and liver disorders (Wu J. et al., 2021). Its significance in liver disorders has received increasing attention, partly because of excess iron levels under various conditions. Therefore, we examined the existing understanding of ferroptosis in different liver diseases. Considering its growing significance in the etiology of several liver disorders, ferroptosis represents a prospective therapeutic target.

In addition, ferroptosis-related molecules and pathways may also have potential clinical relevance as candidate biomarkers for disease evaluation and therapeutic stratification. From a biomarker perspective, ferroptosis-related molecules may reflect key pathological processes in liver diseases, including iron dysregulation, lipid peroxidation, oxidative stress, and impaired antioxidant defense (Ru et al., 2024; Peleman et al., 2024a). Therefore, markers such as GPX4, SLC7A11, ACSL4, TfR1, ferritin-related proteins, and lipid peroxidation products should currently be regarded as candidate biomarkers with potential diagnostic, prognostic, predictive, or pharmacodynamic value, rather than established clinical biomarkers (Zhou et al., 2024; Peleman et al., 2024b). These candidate biomarkers could help evaluate ferroptosis-related tissue injury, disease progression, treatment sensitivity, or target engagement, although their clinical utility requires disease-specific validation and standardized detection methods. The regulatory mechanisms of ferroptosis have been investigated (Sun et al., 2023). Ferroptosis is significantly regulated by the glutathione/glutathione peroxidase 4 (GSH/GPX4) system (Yang et al., 2014). It is also regulated by several key pathways beyond the GPX4-dependent system. The FSP1-Coenzyme Q10 (CoQ10)–NAD(P) H axis reduces CoQ10 to its hydroquinone form, a potent antioxidant that inhibits lipid peroxidation and suppresses ferroptosis independent of GPX4 (Mishima et al., 2022). Additionally, the GTP cyclohydrolase-1 (GCH1)–BH4 pathway, in which GCH1 catalyzes the synthesis of tetrahydrobiopterin (BH4), prevents ferroptosis through a GPX4-independent mechanism (Ma T. et al., 2022). The DHODH–CoQ10 pathway, wherein dihydroorotate dehydrogenase (DHODH) in the mitochondrial inner membrane reduces CoQ10, also contributes to ferroptosis prevention (Mao et al., 2021a). Similarly, FSP1 reduces vitamin K to its hydroquinone form, which suppresses ferroptosis through the non-canonical vitamin K cycle (Mishima et al., 2022). These pathways highlight the complexity and redundancy of ferroptosis regulation and offer promising targets for therapeutic interventions, with potential implications for future clinical translation.

Therefore, this review aims to present ferroptosis in liver diseases as an integrated translational framework. We summarize its core molecular mechanisms, disease-specific roles, potential value as a source of candidate biomarkers, and therapeutic implications. By connecting mechanistic biology with biomarker development and clinical translation, this review highlights the relevance of ferroptosis to precision medicine in liver diseases.

## 2 Molecular basis and clinical relevance of ferroptosis in liver diseases

### 2.1 Overview of ferroptosis and its relevance to liver diseases

Cells are the basic organizational units of life. Thus, cell division, proliferation, functional characteristics, and ultimately, cell death control cell fate and function. As most living organisms rely on oxygen as the last electron acceptor in redox-based metabolic processes, the cellular response to oxidative stress is a crucial determinant of cellular destiny (Jiang et al., 2021). Lipid peroxidation of membrane bilayer components regulates cell destiny, and a substantial buildup of lipid peroxides leads to cell death via ferroptosis (Stockwell et al., 2017; Kim et al., 2023; Dixon et al., 2012; Ma X. et al., 2022). Widespread lipid peroxidation leads to cell death through a unique pathway known as ferroptosis (Stockwell et al., 2017; Dixon et al., 2012). Erastin is a novel substance that destroys RAS-expressing cancer cells. However, the cells die differently than previously observed. Their nuclear morphology does not change, DNA is not broken, caspase is not activated, and caspase inhibitors cannot reverse this process (Dolma et al., 2003; Yagoda et al., 2007). Iron-chelating compounds can prevent this cell death pattern, and RSL3 can initiate the same death pathway (Yagoda et al., 2007; Yang and Stockwell, 2008). This iron-dependent death is called “ferroptosis” (Dixon et al., 2012; Zhao T. et al., 2022).

### 2.2 Molecular mechanisms underlying ferroptosis and their biological significance

A complex network, including lipid metabolism, iron homeostasis, and the oxidative-reductive system, controls ferroptosis (Sun et al., 2023).

#### 2.2.1 The System Xc^−^/GSH/GPX4 regulatory axis

The crucial upstream node of the system Xc^−^/GSH/GPX4 axis has been studied extensively. System Xc^−^, a Cys2/Glu antiporter upstream of this axis, is composed of the regulatory subunit 4F2 (4F2hc)/solute carrier family 3 member 2 (SLC3A2) and the catalytic subunit xCT/solute carrier family 7 member 11 (SLC7A11); SLC7A11 is the primary active functional subunit (Su et al., 2022). SLC7A11 expression is an adaptive mechanism triggered by several stressors, such as metabolic stress, oxidative damage, amino acid deprivation, and genotoxic stress; it facilitates cell survival by reestablishing redox equilibrium (Koppula et al., 2021). System Xc^−^, which imports Cys by substituting internal glutamate (Glu) for extracellular cystine (Cys), is inhibited by high extracellular Glu concentrations (Luo et al., 2021). It mediates the uptake of cystine, which is used for GSH production and is converted into cysteine by thioredoxin reductase 1 and/or GSH (Liu et al., 2020a). GSH is the primary antioxidant in human cells, and cysteine slows down GSH production. Therefore, lowering intracellular cysteine and GSH levels may affect GPX4 activity, potentially resulting in ferroptosis. Erastin and its analogs disrupt system Xc^−^. This may cause endoplasmic reticulum stress, glutathione depletion, cysteine deficiency, and cell death (Xiang et al., 2023).

GSH is the primary cofactor of the system Xc^−^/GSH/GPX4 axis. This fundamental antioxidant is synthesized through the condensation of glutamic acid (Glu), cysteine (Cys), and glycine (Gly) (Wang H. et al., 2023). GSH, the principal cofactor of GPX4, functions as an electron donor or acceptor via the interconversion of G-SH and GS-SG. Thus, it can combat oxidative stress. Erastin lowers GSH levels and causes a morphologically similar type of cell death in sensitive cells owing to GPX4 depletion. Therefore, its use demonstrates a direct relationship between GSH and ferroptosis (Pierzynowska et al., 2021). Ferroptosis is typically induced by inhibiting GSH production (Wang H. et al., 2023). Furthermore, GPX4 is primarily responsible for GSH function in ferroptosis. GPX4 may reduce phospholipid hydroperoxide (PLOOH) to the appropriate hydroxyl derivatives. G-SH may decrease -SeOH and further activate GPX4, thereby releasing GS-SG and preventing GPX4 inactivation. In the catalytic cycle of GPX4, PLOOH oxidizes -SeH (the primary active group of GPX4) to selenic acid (-SeOH) (Su et al., 2022). Many cancer cells undergo ferroptosis owing to GSH depletion and liver malignancy (Badgley et al., 2020; Guo et al., 2023; Xu et al., 2023). Thus, inhibition or antagonization of GSH synthesis can confer an effect similar to that of GPX4 inactivation (Stockwell et al., 2017; Li F. J. et al., 2022; Xue et al., 2025).

The primary downstream antioxidant in the system Xc^−^/GSH/GPX4 axis is GPX4. This phospholipid peroxidation inhibitor serves as the foundation of antioxidant defense; it is the only GSH peroxidase used for intracellular lipid peroxide reduction (Lam et al., 2023). GPX4 prevents ferroptosis by inhibiting the lipid peroxidation chain reaction and converting harmful lipid peroxides into harmless lipid alcohols (Liu X. et al., 2022). GSH provides electrons for GPX4 to reduce lipid peroxides. Hence, low GSH levels lead to decreased GPX4 efficacy. Lipid peroxides consequently accumulate, leading to ferroptosis. The direct or indirect inhibition of GPX4 by reducing GSH synthesis can induce iron sagging. This offers more opportunities to target the GSH-GpX4 axis.

Typical regulatory pathways of GPX4 involve several mechanisms. First, the upregulation of miR-324-3p expression or downregulation of the expression of the androgen receptor (AR) and KIF20A contributes to GPX4 modulation. Secondly, pentane diterpenes inhibit GSH biosynthesis, thereby affecting GPX4 function. Third, ferroptosis inducers, such as erastin and sorafenib, inhibit cysteine uptake. The upregulation of miR-375 and ATF3 expression also regulates GPX4 expression. These pathways highlight the complex regulation of GPX4, which plays a central role in the control of ferroptosis, and offers potential therapeutic targets for modulating this cell death pathway (Zhang C. et al., 2022).

The system Xc^−^/GSH/GPX4 axis plays a critical role in ferroptosis inhibition, particularly in the treatment of solid tumors. Therefore, the mechanisms underlying this axis must be elucidated to facilitate the development of targeted therapies (Li F. J. et al., 2022). The activity of this system is primarily regulated by SLC7A11 (Jyotsana et al., 2022). A decrease in SLC7A11 expression impairs systemic activity, leading to oxidative stress-induced ferroptosis (Yan et al., 2023), whereas SLC7A11 overexpression enhances resistance to ferroptosis (Yan et al., 2023; Huang et al., 2024). Therefore, regulation of SLC7A11 expression is crucial for overcoming ferroptosis resistance. SLC7A11 expression is tightly regulated by multiple mechanisms, including transcriptional and post-transcriptional regulation. These mechanisms involve various pathways that maintain cellular homeostasis, such as p53-mediated transcriptional suppression, NRF2-mediated positive regulation, BAP1-mediated epigenetic repression, and non-coding RNA pathways (Jiang et al., 2015; Zhang Y. et al., 2018; Feng et al., 2021).

Activating transcription factor 4 (ATF4) and nuclear factor erythroid 2-related factor 2 (Nrf2) primarily promote the transcription of SLC7A11 during stress (He F. et al., 2023; Wang D. et al., 2024). mRNA translation induces ATF4 expression under stressful conditions such as viral infection. In the absence of amino acids such as cystine, cells employ the general control non-repressor 2-eukaryotic initiation factor signaling pathway to encourage the translation of ATF4. This promotes the transcription of genes that are involved in the stress response and amino acid metabolism, such as SLC7A11, to mediate amino acid starvation (Li S. et al., 2022; Hayes et al., 2025; Ling et al., 2023). SLC7A11 protects cells from ferroptosis induced by cystine deprivation. Similarly, ATF4 increases tumor angiogenesis by stimulating SLC7A11 transcription (He F. et al., 2023). Nrf2 also stimulates the transcription of SLC7A11 to regulate antioxidant responses. In the absence of stress, Kelch-like ECH-associated protein 1 (Keap1) mediates proteasomal degradation and maintains low Nrf2 levels (Chen et al., 2024). Nrf2 releases the inhibition of Keap1 and activates the transcription of cellular protective genes, such as SLC7A11, by dimerizing with members of the small Maf family and binding to antioxidant response elements in the regulatory region of the cell defense enzyme gene (Ulasov et al., 2022). Downregulation of KEAP1 expression triggers the NRF2–KEAP1 pathway, thereby upregulating SLC7A11 expression to prevent ferroptosis (Shao et al., 2021).

GSH, an antioxidant, prevents ferroptosis. GSH abundance is influenced by both its production and degradation. Glutamate cysteine ligase (GCL) and glutathione synthetase (GSS) continually generate GSH. The rate-limiting step in GSH production is GCL activity (Ma H. et al., 2022). GCL, a heterodimer required for GSH synthesis, is formed by the combination of the GCL modifier (GCLM) and GCL catalytic (GCLC) subunits (Bajic et al., 2019). The genetic suppression of GCLC exacerbates ferroptosis induced by metabolic stress, such as cystine deprivation. GSS catalyzes the conversion of γ-glutamylcysteine to G-SH when GCL binds to cysteine and gluteine (Shi et al., 2023). Therefore, those that block system Xc^−^ activity via Cys depletion may also inhibit GSH production (Li F. J. et al., 2022; Kang et al., 2021). GSH depletion directly affects the function of GPX4, as Cys is an essential limiting amino acid for intracellular GSH production (Zhao J. et al., 2022).

During mammalian ferroptosis, PLOOHs are directly reduced by the antioxidant enzyme GPX4. Selenocysteine (Sec), the twenty-first amino acid, is necessary for its redox action and is incorporated into GPX4 through a specialized translational mechanism involving selenocysteine tRNA maturation (Friedmann Angeli and Conrad, 2018). As a key downstream antioxidant enzyme of the system Xc^−^/GSH/GPX4 axis, GPX4 is often targeted during the development of ferroptosis inducers (Lee and Roh, 2025a). Selenium availability, a possible limiting factor in vulnerability to ferroptosis, modulates GPX4 activity. Selenium is provided via the mevalonate route for GPX4 maturation. However, statins can block this pathway, consequently impairing selenocysteine tRNA synthesis and suppressing GPX4 activity; this enhances susceptibility to ferroptosis (Ingold et al., 2018; Chen et al., 2025). In addition to selenium, the mevalonate pathway produces isopentenyl pyrophosphate, which facilitates GPX4 production (Wu S. et al., 2021). Furthermore, because GSH is a primary cofactor of GPX4, GPX4 may also be indirectly modulated by its regulation.

#### 2.2.2 Iron metabolism in ferroptosis

Iron can donate and acquire electrons, changing from a soluble ferrous form (Fe^2+^) to an insoluble ferric form (Fe^3+^) (Heming et al., 2011). This fundamental component of the human body is involved in the production of numerous significant proteases (Liu X. et al., 2022). Thus, its metabolism, particularly its role in tumorigenesis and cancer progression, has been a key focus in cancer research in the last decade (Bu and Wang, 2025; Teh et al., 2024; Zhang et al., 2024). Numerous biological functions such as oxygen transport, ATP generation, DNA biosynthesis, and cell division depend on Fe. Therefore, iron plays a critical role in DNA synthesis, energy transport, erythropoiesis, and cell signaling (Lei et al., 2021).

Iron homeostasis must be maintained to prevent ferroptosis. Iron intake, export, storage, and utilization affect the intracellular labile iron pool (LIP) (Sun et al., 2023). The transferrin/transferrin receptor (TF/TFR) system is implicated in ferroptosis and is the primary regulator of cellular iron absorption (Lee and Roh, 2025b).

Divalent metal transporter 1 (DMT1), which facilitates iron transport via acidified endosomes and intestinal iron absorption, is one of many proteins that synergistically facilitate iron transport (Morgan and Oates, 2002). In contrast, ferroportin (FPN) is the only exporter of membrane iron (Pasquadibisceglie et al., 2023). Cells take up iron from the iron-binding serum protein transferrin (Tf) and its receptor transferrin receptor 1 (TfR1) (Pasquadibisceglie et al., 2023); ferritin, an intracellular protein that stores iron; and hepcidin, a peptide hormone that circulates and regulates the breakdown of FPN to maintain iron balance (Joachim and Mehta, 2022). Under physiological conditions, the body receives 100% of its iron from food. It is mostly absorbed by the villi of mature enterocytes in the proximal portion of the small intestine, where the low pH maintains its solubility and enables absorption (Morgan and Oates, 2002). Before its uptake by DMT1 at the enterocyte apical membrane, most dietary iron must be converted from the ferric (Fe^3+^) to the ferrous (Fe^2+^) form by reductases such as duodenal cytochrome B (Kumar and Brookes, 2020). In addition, hepatocytes and macrophages are other sources of plasma iron. Macrophages are the greatest iron storage site, whereas hepatocytes cause senescent red blood cells to release iron derived from heme (Ganz, 2025; Nemeth and Ganz, 2021).

Differential plasma Tf binds to TfR1, which is expressed on the membrane of almost all cells, to transport iron into the cells. The Tf–TfR1 complex is internalized via receptor-mediated endocytosis (Paul et al., 2017). Tf undergoes transformation with the aid of the ferrireductase STEAP3, and Tf-bound ferric iron is reduced inside the highly acidified endosome, consequently releasing iron from Tf. DMT1 facilitates iron transport into the cytoplasm via the endosomal membrane. The needs of cells determine intracellular iron levels. For example, iron may be transported directly to areas of utilization, such as mitochondria (Gutowska et al., 2022). If not, ferritin would be used to sequester it in a non-toxic form, creating an iron store for later usage.

Under pathological conditions, a portion of Fe^2+^ is transported to the extracellular matrix through ferritin, whereas DMT1 carries the remaining ferritin (Fn) and the cytoplasmic LIP. A significant amount of Fe^2+^ accumulates in the LIP, generating excessive hydroxyl radicals. This leads to a sudden increase in reactive oxygen species (ROS), resulting in lipid peroxidation and ultimately, ferroptosis (Lee and Roh, 2025b; Li et al., 2023a; Zhong et al., 2025). Thus, intracellular iron excess induced by aberrant iron metabolism is a prerequisite for ferroptosis. Several genes and proteins that maintain iron homeostasis control the susceptibility of cells to ferroptosis (Liu X. et al., 2022). Tumor cells store more intracellular iron than normal cells to support their development and proliferation. However, this extra iron may also increase vulnerability to ferroptosis (He Y. et al., 2023). The regulation of iron metabolism can induce ferroptosis in tumor cells. In particular DHA enhances LIP and downregulates DMT1 and LCN2 expression to regulate the iron metabolism pathway (Zhang C. et al., 2022).

#### 2.2.3 Lipid peroxidation as the execution stage of ferroptosis

ROS readily oxidize polyunsaturated fatty acids (PUFAs), which are involved in the formation of phospholipids in cell membranes. This results in the production of considerable amounts of lipid peroxides. Ferroptosis results from the destruction of the normal structure and function of cell membranes by lipid peroxides and their metabolites. Lipid peroxidation can be initiated enzymatically or non-enzymatically; free radicals catalyze non-enzymatic lipid peroxidation (Anthonymuthu et al., 2016).

ROS initiate a chain reaction driven by free radicals, leading to non-enzymatic lipid peroxidation. They remove hydrogen atoms from PUFAs to create lipid radicals that form peroxyl radicals upon reacting with oxygen. These peroxyl radicals continue to react, resulting in the accumulation of lipid peroxides (Do and Xu, 2024). The most chemically reactive species of activated oxygen is the hydroxyl radical (OH-). This highly mobile, water-soluble form of ROS may initiate lipid peroxidation (Wang and Kang, 2021). The main sources of hydroxyl radical production are Fenton and Fenton-type reactions, which primarily involve the interaction of hydrogen peroxide (H_2_O_2_) with a transition metal, such as free ferrous labile iron (Fe^2+^). The first step in non-enzymatic lipid peroxidation is the creation of a carbon-centered lipid radical, which is produced when a hydroxyl radical removes a hydrogen atom from PUFAs (Tang et al., 2021). Lipid peroxyl radicals are generated when lipid radicals interact with molecular oxygen (Zepeda et al., 2018). A new lipid radical chain reaction is initiated when lipid radicals bind oxygen to form lipid peroxyl radicals (LOO). These subsequently release hydrogen from adjacent PUFAs to form lipid hydroperoxides (LOOH) and radicals (Liu L. et al., 2022). In the presence of ferrous iron, lipid hydroperoxide is transformed into an alkoxy radical, which then binds a nearby PUFA to initiate a new chain reaction of lipid radicals (Valgimigli, 2023; Yu et al., 2021). This auto-amplifying process, which is catalyzed by iron and oxygen, may result in membrane disintegration and cell death when the regulators of lipid peroxidation fail (Mortensen et al., 2023). Chain-breaking lipophilic antioxidants neutralize lipid radicals by donating electrons, which is their main non-enzymatic defense mechanism (Niki, 2014). However, the two radicals may react to create stable nonradical molecules at high concentrations of radicals (Valgimigli, 2023).

Activity of the lipoxygenase (LOX) family regulates enzymatic lipid peroxidation. LOXs, which contain non-heme iron, catalyze the deoxygenation of esterified and free PUFAs to produce various lipid hydroperoxides (Deng et al., 2022). The two most prevalent PUFAs in mammalian cells are linoleic acid (LA) and arachidonic acid (AA); these act as substrates for LOXs (Ma S. et al., 2022). For example, acyl-CoA synthetase long-chain family member 4 (ACSL4) ligates AA to CoA to generate AA-CoA, which is then incorporated into phosphatidylethanolamine by lysophosphatidylcholine acyltransferase-3 (LPCAT3) to form AA-PE. Subsequently, LOXs oxidize AA-PE to HpETE-PE (Doll et al., 2017; Kagan et al., 2017). The accumulation of oxidized phospholipids induces lipid peroxidation, ultimately triggering ferroptosis (Jiang et al., 2021; Kagan et al., 2017). Consistent with a causal role for LOXs, pharmacologic inhibition or genetic knockdown of LOX suppresses ferroptosis in several cell types (Wenzel et al., 2017; Shintoku et al., 2017). In addition to functioning as a lipophilic radical trap, vitamin E can also directly inhibit 15-LOX, which may account for its strong anti-ferroptotic activity (Kagan et al., 2017; Hinman et al., 2018).

Free-radical lipid autoxidation proceeds in three canonical phases: initiation, propagation, and termination (Valgimigli, 2023; Porter et al., 1995). During initiation, hydrogen abstraction or one-electron oxidation generates a carbon-centered lipid radical (L•), which can be triggered by exogenous physical/chemical initiators (e.g., UV or ionizing radiation) or endogenous ROS-producing enzymatic systems (Valgimigli, 2023). During propagation, molecular oxygen rapidly binds L• to form a lipid peroxyl radical (LOO•). LOO• subsequently abstracts a hydrogen atom from a neighboring lipid (LOO• + LH → LOOH + L•), thereby regenerating L• and sustaining the chain reaction (Valgimigli, 2023; Porter et al., 1995). Termination occurs when chain-breaking lipophilic antioxidants such as vitamin E donate a hydrogen atom to LOO•, yielding the corresponding tocopheroxyl radical and halting propagation. Alternatively, radical–radical coupling or disproportionation converts reactive species into non-radical products (Valgimigli, 2023; Niki, 2014; Augustine et al., 2020).

Ferroptosis cannot occur without PUFAs, which are the source of oxidative lipid degradation. The number of accessible lipid peroxidation sites and, thus, sensitivity to ferroptosis, are determined by PUFA availability (Mbah and Lyssiotis, 2022). ROS react with lipid membrane PUFAs to create LROS, resulting in lipid peroxidation. Intracellular overproduction of ROS leads to oxidative stress, which damages proteins and DNA, ultimately resulting in cell death (Beider et al., 2020). Oxidative stress facilitates the accumulation of reactive aldehydes resulting from lipid peroxidation and is linked to cardiovascular diseases. This may lead to an increase ROS/RNS production and/or a decrease in the antioxidant defense (Gallelli et al., 2018).

**FIGURE 1:**
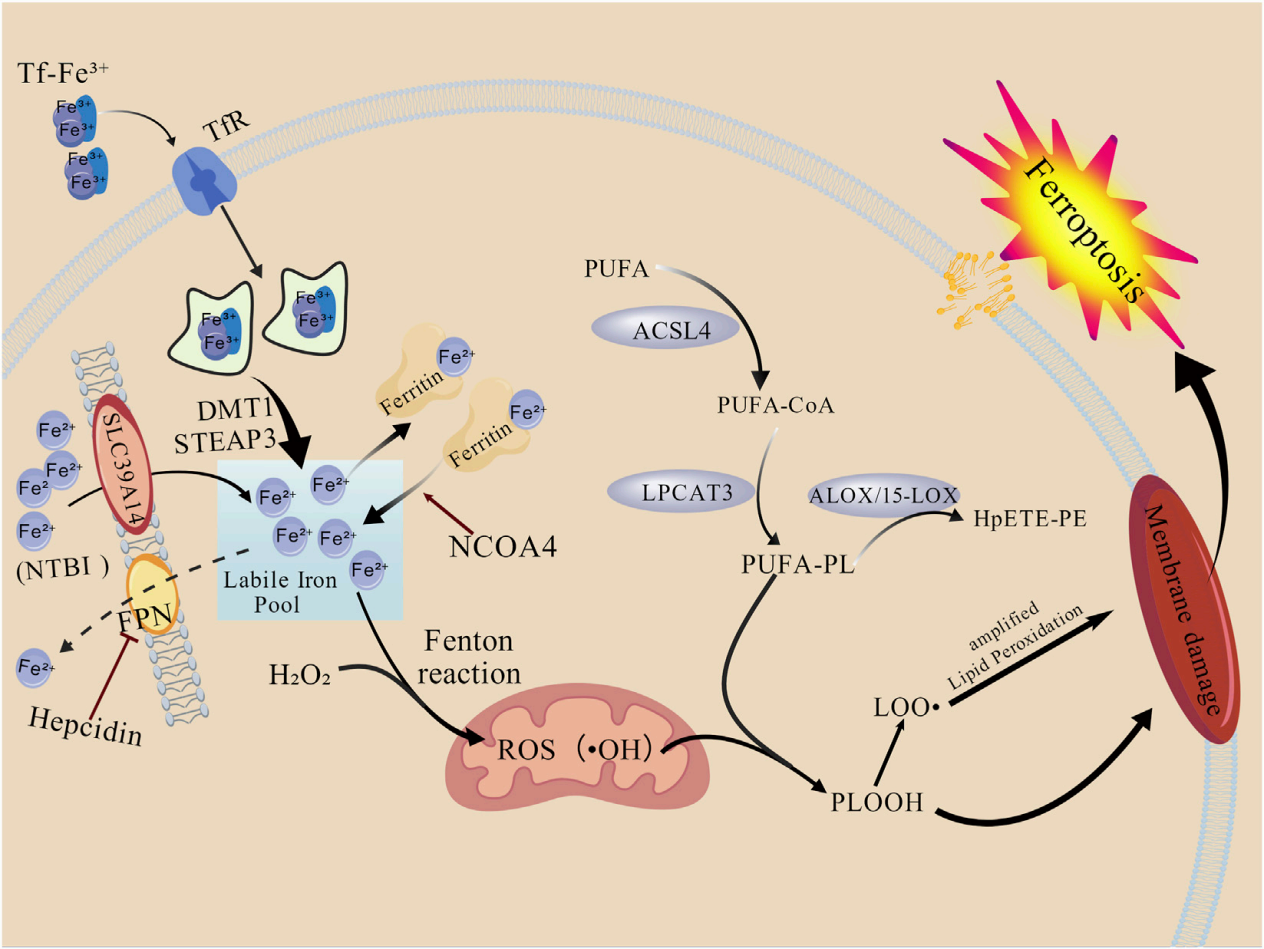
Iron metabolism, ROS generation, and lipid peroxidation in ferroptosis. This diagram summarizes the major biochemical events that drive ferroptotic cell death. Dysregulated iron uptake through Tf/TfR1, DMT1, STEAP3, and SLC39A14 increases the labile iron pool, whereas ferritin, ferroportin, and hepcidin regulate iron storage and export. Excess Fe^2+^ promotes the Fenton reaction and generates ROS, which initiate oxidative stress and lipid peroxidation. Lipid peroxidation occurs through both enzymatic and non-enzymatic mechanisms. The ACSL4–LPCAT3–ALOX/15-LOX axis converts PUFA-containing phospholipids into oxidized lipid species such as HpETE-PE, whereas ROS-driven radical chain reactions generate PLOOH and LOO•. The accumulation of oxidized phospholipids damages cellular and mitochondrial membranes, thereby promoting ferroptotic cell death.

Regulation of the lipid metabolism arm of ferroptosis centers on two nodes: (i) an ARF6-ACSL4 axis loss of ARF6 derepresses ACSL4, enriches PUFA-phospholipids, and heightens ferroptosis sensitivity (Ye et al., 2020); and (ii) miR-522-mediated suppression of lipoxygenases (ALOX15), which reduces lipid-ROS accumulation and blunts ferroptosis in tumor cells (Zhang et al., 2020). These molecular events are summarized in Figure 1, which illustrates how dysregulated iron uptake and storage, ROS production through the Fenton reaction, and ACSL4–LPCAT3–ALOX/15-LOX-mediated lipid peroxidation converge to drive membrane damage and ferroptotic cell death.

### 2.3 Key regulatory pathways of ferroptosis and their translational implications

#### 2.3.1 System Xc^−^ inhibition induces ferroptosis

The phospholipid bilayer has a large distribution of amino acid antitransporters, known as the Xc^−^ system. The two subunits that make up this heterodimer are SLC7A11 and SLC3A2, which are components of the crucial cellular antioxidant system (Zhang J. J. et al., 2021).

Several small-molecule inducers may cause ferroptosis (Lu et al., 2017). Pharmacologic induction of ferroptosis suppresses cancer cell growth in both RAS-dependent (e.g., erastin-sensitive) and RAS-independent contexts (e.g., direct GPX4 inhibition), underscoring the genetic heterogeneity of ferroptotic responses (Yang et al., 2014; Stockwell et al., 2017; Dixon et al., 2012). The principal mechanism involves blockade of the cystine/glutamate antiporter (system Xc^−^) with prototypical agents, including erastin and the clinically used anti-inflammatory sulfasalazine (Sato et al., 2018; Dixon et al., 2014; Wu and Zhu, 2025).

The system Xc^−^ antiporter (SLC7A11/SLC3A2) uses 1:1 stoichiometry to convert extracellular cystine into intracellular glutamate (Lewerenz et al., 2013). Imported cystine is reduced to cysteine, the rate-limiting precursor for GSH synthesis, via GCLC/GSS, thereby supporting the cellular antioxidant capacity (Jyotsana et al., 2022; Lewerenz et al., 2013). GSH serves as an essential cofactor for GPX4 to reduce lipid hydroperoxides and restrain ferroptosis (Ursini and Maiorino, 2020; Yang and Stockwell, 2016). Pharmacologic or genetic inhibition of system Xc^−^ diminishes cystine uptake, depletes GSH, compromises GPX4 activity, and induces lipid-ROS accumulation and ferroptotic death (Sato et al., 2018; Gan, 2023; Su et al., 2020; Li J. et al., 2020). Similarly, the inhibition of system Xc^−^ results in intracellular cystine depletion. Several chemical compounds and their derivatives may block system Xc^−^. For example, the classical oncogenic chemical erastin, one of the first known ferroptosis inducers, blocks system Xc^−^ (Gong et al., 2022).

#### 2.3.2 p53-mediated regulation of ferroptosis

More than half of all human malignancies are characterized by mutations or inactivation of p53, a vital tumor suppressor encoded by *TP53* (Wu et al., 2023). The tumor-suppressive effect of p53 is attributed to its conventional mechanisms: the induction of apoptosis, senescence, and cell-cycle arrest (Chen, 2016). However, by controlling ferroptosis, the non-canonical roles of p53 that govern cellular metabolism and redox equilibrium also aid in tumor suppression. For example, p53 represses SLC7A11 to limit cystine uptake and sensitizes cells to ferroptotic death. In addition, it can engage an ALOX12-dependent lipid peroxidation pathway (Jiang et al., 2015; Chu et al., 2019). Conversely, p53 can restrain ferroptosis under basal or modest oxidative stress (e.g., *via* p21 induction during cystine starvation or by blocking dipeptidyl-peptidase-4 (DPP4) activity at the plasma membrane), whereas it tends to promote ferroptosis under higher oxidative stress. These functional variations indicate a context-dependent rheostat-like behavior (Tarangelo et al., 2018; Xie et al., 2017; Faramarzi et al., 2021).

By inhibiting SLC7A11 transcription under chemical stress and preventing cystine absorption in tumor cells, P53 also increases susceptibility to ferroptosis (Tang et al., 2022). The p53 DNA-binding domain must be acetylated to regulate SLC7A11 expression. p533KR, an acetylation-defective mutant of p53 with three altered lysine subunits (K117/161/162R), does not cause cell cycle arrest, senescence, or apoptosis. Nonetheless, it sensitizes cells to ferroptosis (Yang et al., 2022). p53^3KR^ knock-in mice did not develop early spontaneous tumors, indicating that classical programs (cell-cycle arrest, senescence, and apoptosis) are not strictly required for tumor suppression (Li et al., 2012); together with evidence that p53 promotes ferroptosis by repressing SLC7A11, this supports a role for ferroptosis in p53-mediated tumor control (Jiang et al., 2015; Wang et al., 2016). p53 also controls ferroptosis through its metabolic target spermidine/spermine N1-acetyltransferase (SAT1), a polyamine-catabolic enzyme. SAT1 loss markedly blunts p53-driven ferroptosis. However, its overexpression increases susceptibility to ferroptosis under ROS stress (Ou et al., 2016). Moreover, the African-restricted P47S variant of p53 exhibits impaired ferroptosis, partly due to reduced repression of cystine import programs (e.g., attenuated control of SLC7A11), thereby limiting lipid peroxide accumulation (Leu et al., 2019; Stieg et al., 2023).

p53 can promote or repress ferroptosis depending on the cellular setting (Poschel et al., 2023). Stabilizing wild-type p53 with the MDM2 inhibitor nutlin-3 protects fibrosarcoma, renal cancer, and osteosarcoma cells from system Xc^−^–inhibition–induced ferroptosis by sustaining intracellular GSH levels via the p53-p21 (CDKN1A) axis (Tarangelo et al., 2018; Hassannia et al., 2019). This likely confers a survival advantage under metabolic stress such as cystine deprivation. Furthermore, p53 inhibits erastin-induced ferroptosis by blocking DPP4 activity in colorectal cancer, a mechanism distinct from the previously described proferroptotic role of p53 in other contexts (Jiang et al., 2015; Xie et al., 2017). Additionally, DPP4 interacts with NADPH Oxidase 1 (NOX1) to create a NOX1–DPP4 complex in p53-deficient cells, consequently triggering ferroptosis and plasma membrane lipid peroxidation (Zhang J. et al., 2022).

#### 2.3.3 The NAD(P)H/FSP1/CoQ10 system inhibits ferroptosis

Ferroptosis suppressor protein 1 (FSP1) is a cytoprotective, potent inhibitor of ferroptosis that limits phospholipid peroxide accumulation, even when GPX4 is inactive or deleted (Bersuker et al., 2019; Doll et al., 2019). Therefore, FSP1 loss sensitizes cells to diverse ferroptosis inducers, whereas its overexpression confers resistance across multiple tumor cell lines (Bersuker et al., 2019; Doll et al., 2019). This protein suppresses ferroptosis by catalyzing NAD(P)H-dependent reduction of coenzyme Q (CoQ/ubiquinone) to ubiquinol in non-mitochondrial membranes. This provides a radical-trapping antioxidant system that operates parallel to the canonical GPX4/GSH pathway (Bayir et al., 2020; Lv et al., 2023). FSP1 inhibits ferroptosis by lowering CoQ10 to stop lipid oxidation, and treatment with an FSP1 inhibitor improves cell susceptibility to ferroptosis (Wu J. et al., 2022). GPX4 activity and cellular GSH levels are not necessary for FSP1 function (Lillo-Moya et al., 2021). Thus, a novel surveillance route that differs from the GSH/GPX4 protective pathway was discovered: FSP1/CoQ10/NAD(P)H.

#### 2.3.4 The GCH1/BH4/DHFR system inhibits ferroptosis

GCH1, the rate-limiting enzyme in BH4 biosynthesis, was identified as a ferroptosis-suppressing node. GCH1/BH4 repeatedly emerged as protective in CRISPR activation screens performed under RSL3, erastin-analog treatment, and GPX4 ablation (Jiang et al., 2021; Kraft et al., 2020). GCH1 elevation increases intracellular BH4/BH2, potent radical-trapping antioxidants that curb lipid peroxidation. The GCH1–BH4–phospholipid axis selectively preserves phospholipids bearing two PUFA acyl chains, thereby restraining ferroptosis (Jiang et al., 2021; Kraft et al., 2020). Consistent with this role, GCH1 expression is correlated with ferroptosis resistance across cancer cell lines, and its genetic or pharmacological inhibition sensitizes tumor cells to ferroptosis inducers (Hu et al., 2022). More broadly, cancer-associated metabolic rewiring increases the need for endogenous antioxidants to limit toxic lipid oxidation. BH4 functions as a direct antioxidant that prevents lipid peroxidation/ferroptosis, and it interfaces with CoQ/ubiquinone redox metabolism in membranes (Soula et al., 2020).

#### 2.3.5 Mitochondrial GPX4 and DHODH inhibit ferroptosis

Ferroptosis is a key tumor suppression mechanism (Mao et al., 2021a; Jiang et al., 2021). Two important ferroptosis defense mechanisms involve GPX4 and FSP1 (Bersuker et al., 2019; Doll et al., 2019). N-carbamoyl-L-aspartate, an intermediary in pyrimidine biosynthesis, is acutely depleted and uridine accumulates concurrently when GPX4 inhibitors are used to treat cancer cells (Mao et al., 2021a). Supplementation with dihydroorotate or orotate, the substrate and product of DHODH, respectively, reduced or increased ferroptosis induced by GPX4 inhibition. These effects are more apparent at low GPX4 expression in cancer cells (Zhao et al., 2021). DHODH inactivation induces ferroptosis and considerable mitochondrial lipid peroxidation in cancer cells with low GPX4 expression. In contrast, cancer cells with high GPX4 expression are susceptible to ferroptosis when a DHODH inhibitor is added (Xie et al., 2023). DHODH synergistically works with mitochondrial GPX4 (but independent of cytosolic GPX4 or FSP1) to inhibit ferroptosis in the inner mitochondrial membrane. Specifically, it converts ubiquinone to ubiquinol, an antioxidant that traps radicals and possesses anti-ferroptosis capabilities (Mao et al., 2021a).

Mitochondria are essential organelles in all eukaryotic cells; they regulate energy metabolism, cell survival, apoptosis, and intracellular signaling (Chen et al., 2023). Their primary bioenergetic role is ATP generation *via* oxidative phosphorylation in the electron transport chain, and they are a major source of cellular ROS under many conditions (Murphy, 2009; Brand, 2016). Mammalian cells deploy core antioxidant systems to curb lipid-peroxide damage and ferroptosis, including the GPX4–GSH pathway and the FSP1-CoQ_10_ axis, complemented by the GCH1/BH4-DHFR pathway (Bersuker et al., 2019; Jiang et al., 2021; Soula et al., 2020). In the mitochondria, DHODH couples *de novo* pyrimidine biosynthesis to the respiratory ubiquinone/ubiquinol pool to suppress ferroptosis, thereby linking nucleotide synthesis with mitochondrial redox control (Mao et al., 2021a; Yang et al., 2023).

**FIGURE 2:**
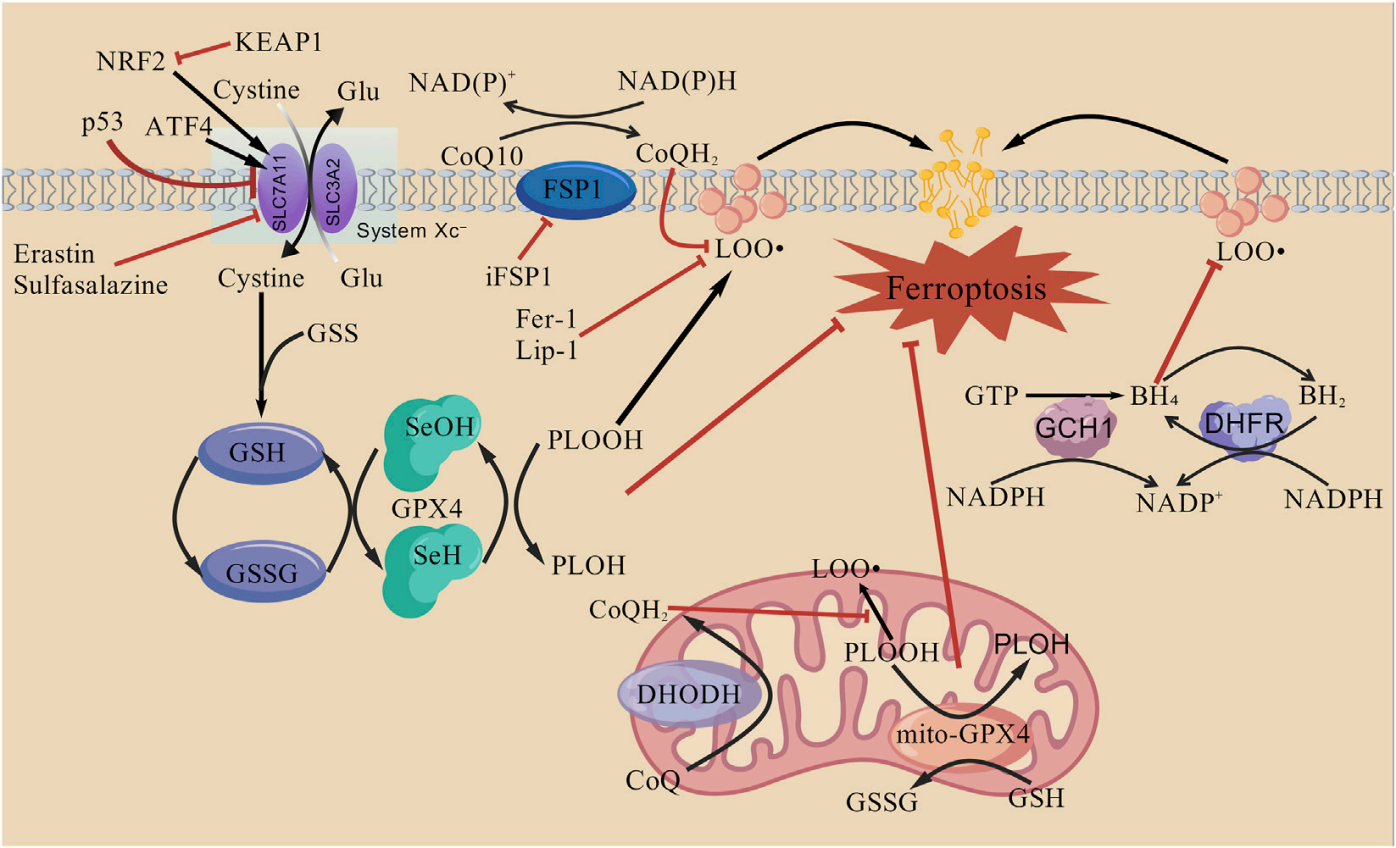
Key ferroptosis-suppressive pathways. This figure illustrates the major antioxidant and metabolic pathways that inhibit ferroptosis. System Xc^−^, composed of SLC7A11 and SLC3A2, mediates cystine uptake for GSH synthesis, thereby supporting GPX4-dependent reduction of PLOOH to PLOH. NRF2 and ATF4 promote SLC7A11 expression, whereas p53 can suppress SLC7A11 and sensitize cells to ferroptosis in specific contexts. The FSP1–CoQ10–NAD(P)H axis reduces CoQ10 to CoQH_2_, which traps lipid radicals at the plasma membrane independently of GPX4. In mitochondria, DHODH reduces CoQ to CoQH_2_ and cooperates with mitochondrial GPX4 to suppress mitochondrial lipid peroxidation. The GCH1–BH4–DHFR pathway maintains BH_4_-dependent antioxidant capacity and further contributes to lipid peroxidation suppression.

The DHODH inhibitor brequinar inhibits the development of GPX4low tumors by inducing ferroptosis (Mao et al., 2021b; Garcia-Bermud et al., 2021). In contrast, combined treatment with brequinar and sulfasalazine, an FDA-approved system Xc^−^ inhibitor, synergistically induces ferroptosis and suppresses GPX4^high^ tumor growth (Liu J. et al., 2022). These results indicate a defensive mechanism for DHODH-mediated ferroptosis in the mitochondria and provide a treatment approach that targets ferroptosis in cancer (Mao et al., 2021b; Garcia-Bermud et al., 2021). Collectively, these pathways form a multilayered ferroptosis-suppressive network, in which system Xc^−^/GSH/GPX4, FSP1–CoQ10, GCH1–BH4–DHFR, and mitochondrial DHODH/mito-GPX4 systems act at different cellular compartments to restrain lipid peroxide accumulation and ferroptotic execution (Figure 2).

### 2.4 Ferroptosis in common liver diseases: mechanistic insights and clinical relevance

**FIGURE 3:**
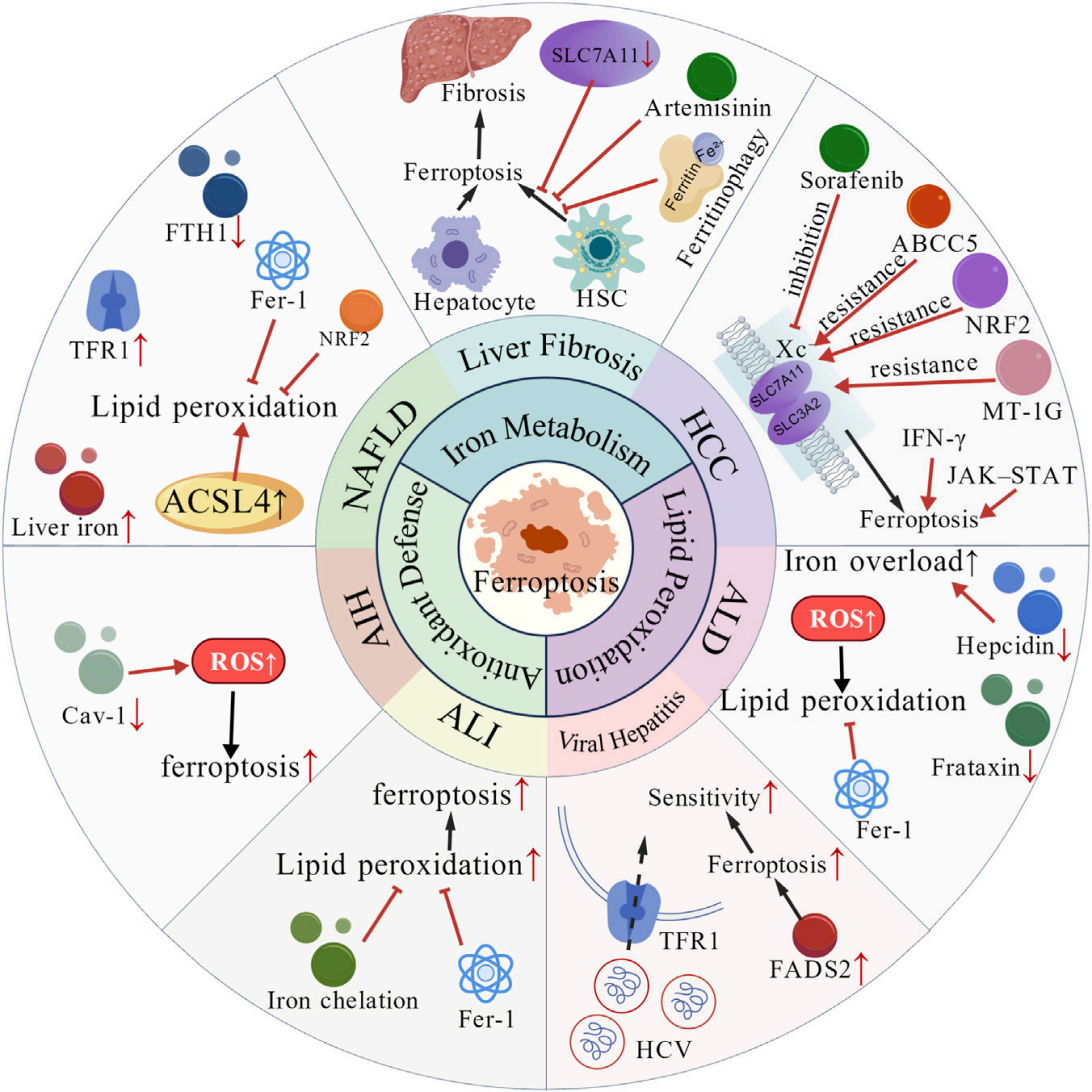
Ferroptotic pathways and disease-specific mechanisms across major liver disorders. This schematic summarizes how core ferroptosis-related processes, including iron metabolism, lipid peroxidation, and antioxidant defense, interact with disease-specific mechanisms in NAFLD/MASLD, ALD, liver fibrosis, acute liver injury, viral hepatitis, autoimmune hepatitis, and HCC. In NAFLD/MASLD, ALD, and acute liver injury, hepatocyte ferroptosis is mainly associated with iron overload, ROS accumulation, and lipid peroxidation. In liver fibrosis, ferroptosis has a context-dependent role: hepatocyte ferroptosis may worsen tissue injury, whereas ferroptosis in activated hepatic stellate cells may contribute to antifibrotic effects. In HCC, ferroptosis induction may promote tumor cell death and modulate treatment sensitivity or resistance. The figure therefore highlights both shared ferroptosis nodes and disease-specific regulatory mechanisms.

Controlled iron-dependent cell death is caused by uncontrolled lipid peroxidation. First characterized in cancer biology, ferroptosis is also implicated in ischemia-reperfusion injury, neurological and hematological disorders, and chronic liver diseases (CLDs). It may play a role in the pathophysiology of ALD, NAFLD, liver fibrosis, and hepatocellular carcinoma (HCC) (Zhou et al., 2022). This section summarizes the current advances linking ferroptosis to these CLDs and outlines the therapeutic strategies that target ferroptotic signaling. As shown in Figure 3, shared ferroptosis-related processes, including iron dysregulation, lipid peroxidation, and impaired antioxidant defense, interact with disease-specific regulators across NAFLD/MASLD, ALD, liver fibrosis, acute liver injury, viral hepatitis, autoimmune hepatitis, and HCC. This figure also highlights the context-dependent effects of ferroptosis, including hepatocyte injury in non-malignant liver diseases, hepatic stellate cell ferroptosis in fibrosis, and tumor cell ferroptosis in HCC. Therefore, Figure 3 should be interpreted as a disease- and cell-type-oriented overview rather than a uniform model, because ferroptosis may produce distinct biological outcomes depending on whether it occurs in hepatocytes, activated hepatic stellate cells, immune cells, or tumor cells.

Ferroptosis is marked by system Xc^−^ dysfunction, disordered iron handling, and aberrant lipid metabolism, culminating in phospholipid peroxidation and cell death across models and patient samples. These convergent features suggest that ferroptosis inhibition to protect the parenchyma or its activation to eliminate malignant cells, may provide tractable therapeutic opportunities for CLDs (Wu J. et al., 2021).

#### 2.4.1 Ferroptosis in NAFLD/MASLD

Following the recent multisociety consensus on steatotic liver disease nomenclature, MASLD and MASH represent the updated terms for NAFLD and NASH, respectively (Rinella et al., 2023). Because much of the existing ferroptosis literature used the terms NAFLD and NASH, these terms are retained when discussing earlier studies, whereas NAFLD/MASLD is used where appropriate to maintain consistency with both historical literature and current terminology.

Alcohol and other liver-damaging factors do not cause NAFLD, a syndrome marked by excessive fat accumulation in liver cells (hepatic steatosis ≥5%) (Xu et al., 2021). Additionally, it is closely associated with insulin resistance and genetic susceptibility. NAFLD includes cirrhosis, nonalcoholic steatohepatitis, and simple fatty liver disease (Park et al., 2017). It has become a leading cause of CLD in Europe and North America, owing to the global increase in obesity and metabolic syndrome. Its prevalence is similarly high in economically developed regions of China (Europe Association for the Study of the, Liver et al., 2024; Wu et al., 2020).

Iron-induced lipid peroxide is one of the main causes of NAFLD, and excess iron is often observed in patients with NAFLD. Furthermore, obesity and insulin resistance, two common traits among patients with NAFLD, are associated with iron imbalance. In addition, Nrf2 controls fatty acid production by suppressing enzyme expression and slows down the development of NASH via the p62/Keap1/Nrf2 axis (Katsuragi et al., 2016). For instance, ferroptosis, rather than necroptosis, is the primary cause of inflammation and cell death in steatohepatitis. More than 90% of patients with NAFLD had high levels of lipid peroxidation indicators in the study by Loguercio et al. (Liu H. et al., 2022).

Studies on NAFLD treatments using targeted ferroptosis have made some progress (Yu and Song, 2024). In the hepatocyte line LO2, Tβ4 effectively prevented cell death caused by palmitic acid (PA) and improved markers related to liver lipid metabolism in rat models of NAFLD induced by a high-fat diet (HFD). The effect of Tβ4 may be increased by combining it with Fer-1. However, the protective effect of Tβ4 may be diminished by either erastin or siRNA interference with GPX4 expression. These findings suggest that Tβ4 protects hepatocytes by preventing the ferroptosis pathway caused by GPX4 depletion (Zhu Z. et al., 2021). The lipid metabolism enzyme enoyl coenzyme A hydratase 1 (ECH1) is essential for mitochondrial fatty acid β-oxidation (Liu B. et al., 2021). ECH1 knockdown can worsen hepatic steatosis, inflammation, and fibrosis in mice. However, Fer-1 intervention may reverse the pathological changes caused by ECH1 knockdown. This suggests that ECH1 shortens the duration of NASH by inhibiting ferroptosis in mice (Zhou et al., 2022).

Traditional Chinese medicines and their constituents have considerably affected the management of NAFLD (Yang et al., 2020; Wu Q. et al., 2022). Iron overload, elevated TfR1 expression, and downregulated FTH1 expression were observed in both PA-/OA-induced HepG2 cells and HFD-induced ApoE^−/−^ mouse models. Ginkgolide B (GB) may reduce iron overload and markedly boost Nrf2 expression (Yang et al., 2020). These findings suggest that GB slows the development of NAFLD by controlling the Nrf2 signaling pathway, which prevents lipid buildup and hepatocyte ferroptosis induced by oxidative stress.

#### 2.4.2 Ferroptosis in ALD

ALD refers to the liver damage caused by long-term excessive alcohol consumption. It first appears as significant steatosis of liver cells and can develop into cirrhosis, liver fibrosis, or steatohepatitis (Jophlin et al., 2024). Severe alcohol misuse can also cause acute alcoholic hepatitis, acute and chronic liver failure, and even death (Jophlin et al., 2024).

Although lipid disorders and liver iron overload are common characteristics of ALD (Li L. X. et al., 2022; Ali et al., 2023), ferroptosis is negatively regulated during the healing of alcohol-induced liver damage. However, researchers have only recently started to investigate its role in ALD (Wu J. et al., 2021). Alcohol treatment led to excessive ROS accumulation and lipid peroxidation in cell cultures and ALD mouse models, which can be rescued by iron removal inhibitors. Furthermore, ferroptosis is the downstream mechanism through which certain genes, such as frataxin, damage the liver upon exposure to high alcohol levels (Liu C. Y. et al., 2020; Luo et al., 2023; Li et al., 2023b).

Hepatic iron overload exacerbates alcohol-related liver damage and is independently linked to worse outcomes, including increased mortality (Ganne-Carrie et al., 2000). Animal studies have further demonstrated that dietary iron and ethanol act synergistically to accelerate oxidative stress, transaminase elevation, fibrosis, and cirrhosis compared with ethanol alone (Tsukamoto et al., 1995). Moreover, the ferroptosis inhibitor ferrostatin-1 (Fer-1) significantly attenuated alcohol-induced liver injury *in vitro* and *in vivo*, supporting an etiological role of ferroptosis in ALD progression (Liu C. Y. et al., 2020; Luo et al., 2023).

The mitochondrial protein frataxin mainly controls oxidative stress reactions and preserves iron homeostasis. Frataxin deficiency leads to increased iron content and lipid peroxidation levels in the mitochondrial LIP, consequently mediating alcohol-induced ferroptosis and contributing to ALD progression (Liu et al., 2020c). Furthermore, intestinal SIRT1 knockout protected mice from alcohol-induced liver inflammation. This effect was attributed to improved iron metabolism, elevated liver GSH levels, and decreased lipid peroxidation (Zhou et al., 2020). These results suggest that a possible treatment approach for ALD may involve the inhibition of ferroptosis in the liver by targeting intestinal SIRT1. Clinical studies have also reported decreased levels of hepcidin in the livers of patients with ALD. This indicates that chronic alcohol consumption may lead to liver iron overload, which consequently induces ferroptosis (Ali et al., 2023). Therefore, the restoration of hepcidin expression or regulation of iron homeostasis through other mechanisms may provide novel avenues for ALD therapy.

#### 2.4.3 Ferroptosis in hepatic fibrosis

Liver fibrosis is a maladaptive wound-healing response to persistent hepatic injury. It is characterized by excessive extracellular matrix deposition, predominantly driven by fibrillar collagen I through the activation of hepatic stellate cells (HSCs) into collagen-secreting myofibroblasts (Kisseleva and Brenner, 2021; Matsuda and Seki, 2020). Major etiologies include alcohol-associated liver disease, NAFLD, and chronic hepatitis B or C viral infections. Crucially, etiology-directed therapies can halt ongoing damage and promote histological fibrosis regression in many patients. These therapies include sustained virological response after direct-acting antivirals in HCV, long-term nucleos(t)ide analogs in HBV, and lifestyle interventions such as alcohol abstinence or weight-loss strategies for ALD/NAFLD (Rockey and Friedman, 2021; Sinclair et al., 2024; Hofer et al., 2023; Verrastro et al., 2023). Despite these advances, no direct antifibrotic drugs have been approved, underscoring an urgent therapeutic need (Friedman and Pinzani, 2022). Fibrosis regression is accompanied by a quantitative and functional reduction of activated HSCs through apoptosis, immune-mediated clearance/senescence, and, in part, reversion toward a less-fibrogenic or quiescent-like state. Experimental and translational studies support HSC deactivation/clearance as a viable antifibrotic strategy (Krizhanovsky et al., 2008; Troeger et al., 2012).

Ferroptosis has become a mechanistic node that connects fibrogenesis and redox metabolism (Lu et al., 2023). Selectively triggered ferroptosis in activated HSCs attenuates experimental liver fibrosis, whereas pathways that suppress HSC ferroptosis (e.g., the MafG/MYH9-LCN2 axis) exacerbate scarring. Conversely, indiscriminate ferroptosis in parenchymal hepatocytes may aggravate tissue injury, highlighting the importance of cell-type–selective approaches (Deng et al., 2024; Wang Y. et al., 2024; Luo et al., 2022; Zhang Z. et al., 2021). These insights suggest that ferroptosis-targeted HSC-selective modulation is a promising antifibrotic strategy that warrants further preclinical and clinical development.

HSCs contain large amounts of iron, which is necessary for ferroptosis (Pan et al., 2021). Liver fibrosis, liver cancer, and CLD are closely associated with ferroptosis (Tan et al., 2022). For example, excessive hepatic iron deposition and ferroptosis exacerbated acetaminophen (APAP)-induced liver fibrosis in mice, which Fer-1 reversed. These results indicate that ferroptosis can promote the development of liver fibrosis (Pan et al., 2021). Ferroptosis is also associated with iron-overload-induced liver injury and fibrosis. Its inhibition mitigates iron overload-related liver injury and scarring. In hepatocyte-specific Tf knockout (Trf-LKO) mice, a high-iron diet or CCl_4_ triggered ferroptosis-dependent liver fibrosis, whereas Fer-1 treatment markedly reversed the fibrotic phenotype. Clinical samples from patients with cirrhosis showed reduced Tf with increased hepatic iron and lipid peroxidation, supporting a protective role for Tf in iron homeostasis and ferroptosis restraint (Yu et al., 2020). Similarly, high-iron feeding induced hepatic ferroptosis and increased Sirius Red-positive collagen deposition; Fer-1 partially rescued iron overload-induced injury (Wang et al., 2017).

Amplified lipid peroxidation and ferroptosis exacerbate APAP hepatotoxicity under iron-overload conditions. Both iron chelation and ferroptosis inhibition attenuate liver damage, which can secondarily aggravate fibrogenic responses if left unchecked (Pan et al., 2021). The induction of ferroptosis in hepatocytes tends to intensify tissue injury and accelerate fibrogenesis, whereas triggering ferroptosis in activated HSCs reduces collagen deposition and inflammatory cell-selective targeting, which is a central prerequisite for therapy (Chen et al., 2022; Wang Y. et al., 2024; Luo et al., 2022).

The direct induction of HSC ferroptosis with celastrol and artesunate/dihydroartemisinin attenuated fibrosis across diverse mouse models (CCl_4_, cholestasis, and dietary models). These mechanisms involved ROS amplification, NCOA4-mediated ferritinophagy, and the ROCK1/ATF3 axis (Zhu Y. et al., 2021). Conversely, xCT/SLC7A11, the cystine/glutamate antiporter that supplies cystine for GSH synthesis, is a nodal regulator; inhibition of SLC7A11 in myofibroblastic HSCs induces ferroptosis and alleviates fibrosis. Contrastingly, non-selective blockade can worsen parenchymal injury, underscoring delivery and cell specificity (Du et al., 2021).

The TF/non-transferrin-bound iron (NTBI)/SLC39A14 uptake pathway is associated with ferroptosis and fibrosis. Trf-LKO mice were hypersensitive to high-iron- or CCl_4_-triggered, ferroptosis-dependent fibrosis and responded to Fer-1 rescue. Thus, limiting NTBI uptake and restoring TF function (potentially alongside ferroptosis inhibitors or iron chelation) are plausible antifibrotic strategies (Jiang et al., 2022). Ferroptosis is a “double-edged sword” that contributes to liver fibrosis and suppresses hepatocyte ferroptosis, while selectively inducing HSC ferroptosis. This represents a bidirectional, mechanistically grounded approach with translational promise.

Several natural compounds can modulate ferroptotic pathways to prevent or attenuate liver fibrosis; encouraging safety signals have been reported in cell and animal models (Lu et al., 2023). Among these, artesunate alleviates CCl_4_-induced fibrosis by triggering ferritinophagy-mediated ferroptosis in activated HSCs. Recent studies further implicate the ROCK1/ATF3 axis in artesunate ferroptotic, antifibrotic activity (Wang Y. et al., 2024; Kong et al., 2019). Artemether confers robust anti-fibrotic effects *in vitro* and *in vivo* by inducing HSC ferroptosis, which stabilizes IRP2 by inhibiting its ubiquitination, thereby increasing intracellular iron and ROS levels. In addition, it requires p53-dependent ferroptosis to suppress HSC activation and CCl_4_-driven scarring (Li Y. et al., 2020; Wang L. et al., 2019). Magnesium isoglycyrrhizinate also exerts antifibrotic activity by inducing HSC ferroptosis-promoting iron and lipid peroxide accumulation, an effect upstream of HO-1 that is abrogated by Fer-1 (Sui et al., 2018).

Beyond artemisinin derivatives, chrysophanol (from rheum rhizome) attenuates HBx-driven HSC activation through ER stress-linked, ferroptosis-dependent, yet GPX4-independent mechanisms, thereby reducing the secretion of profibrotic markers (Kuo et al., 2020). In addition, wild bitter melon (*Momordica charantia*) extract mediates LPS-induced HSC activation and modulates ferroptosis markers (e.g., GPX4 and SLC7A11) toward a pro-ferroptotic pattern in preclinical models, supporting an anti-fibrotic mode of action via ferroptosis (Ho et al., 2021). Collectively, these findings highlight ferroptosis as a tractable therapeutic node for natural product-based antifibrotic strategies, particularly those that selectively trigger ferroptosis in activated HSCs, while sparing hepatocytes. Recent evidence continues to support a cell-type-specific role of ferroptosis in liver fibrosis, involving hepatocytes, activated HSCs, and hepatic macrophages (Zhu et al., 2025). This reinforces the need to distinguish profibrotic ferroptosis associated with parenchymal injury from antifibrotic ferroptosis selectively induced in activated HSCs.

#### 2.4.4 Ferroptosis in acute liver injury (ALI)

ALI is characterized by a rapid decline in liver cell function. It is often caused by drugs, alcohol, ischemia, or viral hepatitis. APAP-induced liver damage can trigger ferroptosis. However, Fer-1 prevented APAP-induced primary hepatocyte death in mice (Wang Y. et al., 2023). Similarly, a mouse model of APAP-induced acute liver failure showed ferroptosis; an increase in lipid peroxides derived from n-6PUFA was observed. These findings suggest that ferroptosis plays a significant role in the development of LPS-induced ALI. Thus, it could be a new therapeutic target for patients with ALI (Liu P. et al., 2020).

#### 2.4.5 Ferroptosis in HCC

The most common type of primary liver cancer is HCC, which has a poor prognosis and high death rate due to frequent recurrence and ineffective treatment (Wang et al., 2022). Ferroptosis has potential applications in cancer treatment (Sanchez et al., 2021).

Alterations in iron metabolism affect HCC pathophysiology (Xie et al., 2023). High iron contents may promote the development of HCC. Thus, a high dietary iron intake can increase the risk of this disease. However, ferroptosis activation may effectively inhibit the development of HCC cells. Therefore, ferroptosis is a novel therapeutic target for HCC.

Sorafenib is the first line of treatment for advanced HCC (Zhang K. et al., 2018). Ferroptosis inhibits the cystine/glutamate antiporter SLC7A11. Thus, it may be the primary mechanism underlying the anticancer effect of this drug. For example, ABCC5, a novel regulator of ferroptosis in HCC cells, can inhibit ferroptosis by stabilizing the SLC7A11 protein, increasing intracellular GSH generation, and reducing the accumulation of lipid peroxidation products. Conversely, downregulation of ABCC5 expression significantly decreased the resistance of carcinoma cells to sorafenib (Huang et al., 2021). MT-1G, a gene regulated by the Nrf2 pathway, is a key factor in the development of sorafenib resistance. Clinical studies reported an increase in serum MT-1G protein levels among patients with HCC who were undergoing sorafenib treatment. MT-1G knockdown accelerated GSH depletion and lipid peroxidation, promoted ferroptosis, and enhanced sorafenib efficacy. These results suggest that MT-1G is a potential therapeutic target for improving treatment outcomes. This gene can sensitize HCC cells to ferroptosis, in addition to serving as a prognostic biomarker (Sun et al., 2016).

In addition to sorafenib, the discovery of medications that target ferroptosis-related pathways for the treatment of HCC has emerged as a research hotspot. Recent studies have highlighted the potential of targeting ferroptosis-related pathways for HCC treatment. For instance, IFN-γ inhibits the cystine/glutamate antiporter system Xc^−^ by activating the JAK/STAT signaling pathway, thereby enhancing the sensitivity of HCC cells to ferroptosis (Yu et al., 2022; Kong et al., 2021). Recent studies have further reinforced the relevance of ferroptosis to HCC treatment resistance, particularly in the context of sorafenib responsiveness, NRF2-related antioxidant defense, and ferroptosis-associated molecular markers such as MT1G and SLC7A11 (Liu R. et al., 2024). These findings support the potential value of ferroptosis-related molecules as predictive biomarkers and therapeutic targets for overcoming drug resistance in HCC.

#### 2.4.6 Ferroptosis in viral hepatitis

Numerous hepatitis viruses, which primarily cause liver lesions, can cause viral hepatitis (Wang M. et al., 2019). Chronic carriers of this virus often have no history of acute illness. However, they can develop cirrhosis (liver scarring), which can cause liver failure, or liver cancer (Choi et al., 2022).

TfR1 plays a major role in iron metabolism within HCV-infected hepatocytes; it facilitates HCV entry into hepatocytes. Its expression can affect intracellular iron content, which is correlated with disease severity. Additionally, fatty acid desaturase 2 (FADS2) determines the sensitivity of cells to ferroptosis and the permissiveness of HCV replication. FADS2 inhibition markedly enhances HCV replication. Similarly, erastin alters the structure of HCV replicase, consequently increasing its vulnerability to antiviral medications that target viral proteases. These findings suggest that modulation of the iron-removal pathway weakens HCV replication (Yamane et al., 2022).

In an *in vitro* study involving human cells infected with the hepatitis A virus 3C protease (3Cpro), lipid peroxidation was accompanied by 3Cpro-induced cell death. This process was effectively inhibited by iron chelation, highlighting the involvement of ferroptosis in virus-induced cell death (Komissarov et al., 2021).

#### 2.4.7 Ferroptosis in autoimmune hepatitis (AIH)

Autoimmune liver disease is a CLD caused by malfunctioning of the immune system (Chen et al., 2020). The disordered immune system attacks liver cells, causing inflammation. In extreme situations, cirrhosis with clinical signs of decompensation, or even the emergence of a malignant illness, may arise from acute or chronic liver failure (Kosuta et al., 2023).

In a ConA triggered Cav-1-deficient mouse model, Cav-1 knockout significantly induced ferroptosis by promoting the accumulation of ConA-induced ROS and reactive nitrogen species. This indicates the potential value of targeting ferroptosis for improving AIH.

### 2.5 Translational and clinical implications of ferroptosis in liver diseases

Given the rapid expansion of ferroptosis research, recent studies increasingly emphasize the need to move from pathway description toward clinically measurable biomarkers, disease-specific validation, and pharmacodynamic monitoring of ferroptosis-targeted interventions (Ru et al., 2024; Zhou et al., 2024; Peleman et al., 2024b). In addition to its mechanistic role in liver disease progression, ferroptosis has increasing translational relevance in the context of therapeutic targeting and the identification of candidate biomarkers. Current evidence mainly supports its value in target discovery, treatment sensitization, and pharmacodynamic evaluation, although most data remain preclinical. Therefore, the clinical translation of ferroptosis-related strategies in liver diseases still requires further validation, as summarized in Table 1.

**TABLE 1:** Clinical utility of ferroptosis inducers and inhibitors in liver diseases.

Category	Mechanism	Examples	Clinical application	Ref. no.
Ferroptosis Inducers	Trigger ferroptosis through excessive iron accumulation and lipid peroxidation	Erastin, Sulfasalazine, System Xc^−^ inhibitors	Emerging therapeutic strategy for liver cancer and liver fibrosis	Wang L. et al. (2019), Choi et al. (2022), Yamane et al. (2022), Komissarov et al. (2021)
GPX4 Inhibition	Inactivation of GPX4 results in lipid peroxidation accumulation, leading to ferroptotic cell death	GPX4 selective inhibitors	Promising approach for cancer treatment, though clinical translation is still under investigation	Dixon et al. (2014), Kosuta et al. (2023)
Nanoparticle Inducers	Enhance ferroptosis induction via nanomaterials that target cancer cells	Nano-ferroptosis inducers	Potential application in nanomedicine for targeted cancer therapy	Hino et al. (2025), Sha et al. (2021)
Ferroptosis Inhibitors	Prevent ferroptosis by chelating iron or inhibiting lipid peroxidation	Deferoxamine (DFO), Ferrostatin-1, Liproxstatin-1	Can be used to protect against liver injury, with ongoing efforts to optimize pharmacokinetics	Qi et al. (2022), Chen et al. (2017), Zheng et al. (2023)
Immune Modulation	Ferroptosis releases DAMPs (e.g., KRAS-G12D, HMGB1) that modulate immune responses	ROS scavengers, Catalase + CAR-T combination	Modulates tumor immunity and enhances antitumor responses	Yang et al. (2016), Salvioni et al. (2019), Yu et al. (2023), Zhang et al. (2025), Scarpellini et al. (2023)

In translational and clinical settings, ferroptosis-related markers may be evaluated using different clinical sample types, including serum or plasma, liver biopsy tissue, tumor tissue, and liquid biopsy-based samples. Circulating samples may be suitable for assessing iron metabolism-related indices, oxidative stress products, and lipid peroxidation-associated metabolites, whereas biopsy or tumor tissues can provide more direct information on cell-type-specific ferroptosis activity. Representative detection platforms include immunohistochemistry or immunofluorescence for key proteins such as GPX4, SLC7A11, ACSL4, FTH1, and TfR1; transcriptomic, proteomic, metabolomic, and spatial omics approaches for ferroptosis-related molecular signatures; and biochemical assays for lipid peroxidation products such as malondialdehyde, 4-hydroxynonenal, and oxidized phospholipids (Ru et al., 2024; Peleman et al., 2024b). From a practical perspective, serum, plasma, and liquid biopsy-based samples may be more suitable for repeated clinical monitoring, whereas liver biopsy or tumor tissue may be more informative for tissue-level, spatial, or cell-type-specific assessment of ferroptotic activity. These approaches may facilitate biomarker discovery, patient stratification, and pharmacodynamic monitoring of ferroptosis-targeted interventions. To provide a clearer overview of their translational relevance, representative ferroptosis-related candidate biomarkers in liver diseases are summarized in Table 2, including their disease context, biological role, potential clinical application, and current evidence level.

**TABLE 2:** Representative ferroptosis-related molecules and candidate biomarkers in liver diseases.

Molecule or indicator	Disease context	Biological role	Current translational category	Potential clinical relevance	Evidence level	Representative references
GPX4	NAFLD/MASLD, ALD, ALI, fibrosis, HCC	Reduces lipid hydroperoxides and suppresses ferroptosis	Therapeutic target and candidate biomarker	May indicate ferroptosis susceptibility, tissue injury, treatment response, or target engagement	Moderate	Ru et al. (2024), Peleman et al. (2024a), Peleman et al. (2024b), Yang et al. (2014)
SLC7A11/system Xc^-^	HCC, fibrosis, NAFLD/MASLD	Supports cystine uptake, GSH synthesis, and GPX4 activity	Therapeutic target and candidate biomarker	May support assessment of ferroptosis resistance, treatment sensitivity, or pharmacodynamic response to ferroptosis induction	Moderate	Ru et al. (2024), Zhou et al. (2024), Peleman et al. (2024b), Jyotsana et al. (2022), He F. et al. (2023), Li S. et al. (2022)
ACSL4	NAFLD/MASLD, ALD, ALI, HCC	Promotes PUFA-phospholipid remodeling and lipid peroxidation	Candidate biomarker and mechanistic regulator	May reflect ferroptosis-prone lipid remodeling, disease activity, or ferroptosis sensitivity	Moderate	Ru et al. (2024), Peleman et al. (2024a), Peleman et al. (2024b), Doll et al. (2017), Ye et al. (2020)
FSP1	HCC and tumor-related contexts	GPX4-independent ferroptosis suppressor via CoQ10 reduction	Therapeutic target and candidate biomarker	May indicate ferroptosis resistance and help identify tumors requiring combination ferroptosis-targeted strategies	Emerging	Ru et al. (2024), Zhou et al. (2024), Mishima et al. (2022), Doll et al. (2019), Bayir et al. (2020), Lv et al. (2023)
DHODH	HCC, mitochondrial ferroptosis-related contexts	Suppresses mitochondrial lipid peroxidation through CoQH_2_ production	Therapeutic target and candidate biomarker	May reflect mitochondrial ferroptosis vulnerability or response to DHODH-targeted interventions	Emerging	Ru et al. (2024), Zhou et al. (2024), Mao et al. (2021a), Yang et al. (2023), Mao et al. (2021b), Garcia-Bermud et al. (2021), Liu X. et al. (2022)
TfR1	HCC, viral hepatitis, ALI, iron-overload injury	Mediates transferrin-bound iron uptake	Candidate biomarker and therapeutic target candidate	May indicate iron uptake, ferroptosis susceptibility, or disease severity in iron-related liver injury	Emerging to moderate	Ru et al. (2024), Zhou et al. (2024), Lee and Roh (2025b), Pasquadibisceglie et al. (2023), Yamane et al. (2022)
Ferritin/FTH1	Fibrosis, NAFLD/MASLD, ALD, ALI	Regulates iron storage; ferritinophagy releases iron for ferroptosis	Candidate biomarker and mechanistic regulator	May reflect iron storage, ferritinophagy activity, and ferroptosis-related tissue injury	Moderate	Ru et al. (2024), Zhou et al. (2024), Peleman et al. (2024b), Joachim and Mehta (2022), Pan et al. (2021), Yu et al. (2020)
Hepcidin	ALD, chronic liver disease, iron-overload injury	Regulates systemic iron homeostasis and ferroportin degradation	Candidate biomarker	May indicate systemic iron dysregulation and risk of iron-driven ferroptotic injury	Moderate	Galy et al. (2024), Lakhal-Littleton and Peyssonnaux (2025), Nemeth et al. (2004), Fisher et al. (2025), Zhou et al. (2024), Joachim and Mehta (2022), Ali et al. (2023)
Lipid peroxidation products	NAFLD/MASLD, ALD, ALI, fibrosis, HCC	Reflect oxidative phospholipid damage	Candidate biomarker	May serve as diagnostic or pharmacodynamic indicators of lipid peroxidation and ferroptosis-associated injury	Moderate	Ru et al. (2024), Peleman et al. (2024a), Peleman et al. (2024b), Anthonymuthu et al. (2016), Valgimigli (2023), Kagan et al. (2017), Liu H. et al. (2022)
GSH/GSSG-related indicators	NAFLD/MASLD, ALD, ALI, HCC	Reflect redox balance and GPX4-supporting antioxidant capacity	Candidate biomarker	May indicate oxidative stress, antioxidant impairment, and response to ferroptosis-modulating interventions	Moderate	Ru et al. (2024), Zhou et al. (2024), Peleman et al. (2024b), Yang et al. (2014), Wang H. et al. (2023), Ma T. et al. (2022), Bajic et al. (2019), Shi et al. (2023), Kang et al. (2021), Zhao T. et al. (2022), Sato et al. (2018)

Evidence levels are assigned qualitatively based on representative mechanistic, preclinical, translational, and clinical evidence discussed in the corresponding sections of this review. “Moderate” indicates relatively consistent mechanistic and translational support but limited disease-specific clinical validation; “Emerging” indicates evidence mainly derived from mechanistic or preclinical studies; and “Emerging to moderate” indicates evidence stronger than early experimental observations but not yet sufficiently validated in independent clinical cohorts. These molecules should be interpreted as candidate biomarkers rather than established clinical biomarkers unless validated in disease-specific clinical cohorts.

Therapeutic targets and biomarkers should be distinguished conceptually. A ferroptosis-related therapeutic target is a molecule or pathway that can be modulated to alter ferroptotic activity, whereas a biomarker is a measurable indicator that may support diagnosis, prognosis, treatment selection, or pharmacodynamic monitoring. Some molecules, such as GPX4, SLC7A11, ACSL4, or TfR1, may have dual relevance, but experimental targetability does not necessarily indicate established clinical biomarker utility. Therefore, candidate biomarkers in this review are interpreted according to measurability, disease context, and evidence level, whereas therapeutic targets are discussed according to their functional role and potential druggability (Ru et al., 2024; Zhou et al., 2024).

The strength of biomarker-related evidence differs across liver diseases. At present, HCC and liver fibrosis have relatively stronger mechanistic and translational support (Zhou et al., 2024; Hino et al., 2025). In HCC, ferroptosis-related molecules have been associated with tumor progression, treatment sensitivity, sorafenib resistance, and potential pharmacodynamic monitoring. In liver fibrosis, studies have linked ferroptosis to hepatic stellate cell activation, hepatocyte injury, iron handling, and antifibrotic responses, providing a clearer basis for candidate biomarker development. By contrast, evidence in viral hepatitis, autoimmune hepatitis, and acute liver injury remains more limited and is largely based on experimental or preclinical models. For NAFLD/MASLD and ALD, accumulating evidence supports the involvement of iron overload, oxidative stress, lipid peroxidation, and GPX4-related pathways, but disease-specific clinical biomarker validation remains insufficient (Peleman et al., 2024a; Peleman et al., 2024b). Therefore, ferroptosis-related biomarkers should be interpreted according to disease context, evidence level, and translational readiness.

From a precision medicine perspective, ferroptosis-related biomarkers may help translate molecular mechanisms into individualized clinical applications. These biomarkers could support patient stratification by identifying subgroups with high ferroptotic activity or ferroptosis-related treatment resistance, inform treatment selection by indicating whether ferroptosis induction or inhibition is more appropriate, contribute to risk prediction by reflecting progressive liver injury, fibrogenic activity, or tumor aggressiveness, and enable treatment-response monitoring through dynamic assessment of target engagement or pharmacodynamic effects. For example, a ferroptosis-resistant molecular profile in HCC, such as enhanced antioxidant defense or altered SLC7A11/GPX4-related signaling, may help identify patients who require combination therapeutic strategies, whereas ferroptosis-related markers of hepatocyte injury or hepatic stellate cell activation may help identify patients at higher risk of fibrosis progression. Such applications remain exploratory but provide a rational framework for integrating ferroptosis biology into precision medicine strategies for liver diseases (Zhou et al., 2024; Peleman et al., 2024b).

Ferroptosis should be interpreted as a context-dependent and cell-type-specific process in liver diseases. In HCC, ferroptosis induction may be beneficial by promoting tumor cell death, enhancing treatment sensitivity, and overcoming therapeutic resistance. In contrast, excessive hepatocyte ferroptosis may aggravate parenchymal injury in NAFLD/MASLD, ALD, and acute liver injury through lipid peroxidation and oxidative stress (Peleman et al., 2024a; Peleman et al., 2024b). Liver fibrosis further illustrates this double-edged role: ferroptosis in activated hepatic stellate cells may exert antifibrotic effects, whereas hepatocyte ferroptosis may worsen tissue injury and fibrogenesis (Hino et al., 2025). Thus, ferroptosis-targeted strategies should be tailored to disease type, disease stage, and affected cell population.

#### 2.5.1 Therapeutic induction of ferroptosis

Numerous ferroptosis inducers, along with ferroptosis-related genes and pathways, have been identified. In particular, excessive lipid peroxides and intracellular iron overload are fatal factors in ferroptosis (Sha et al., 2021). Therefore, ferroptosis inducers are being explored as a potential therapeutic strategy, particularly in malignancy, and may also provide useful tools for evaluating ferroptosis-related responses in disease models.

##### 2.5.1.1 System Xc^*-*^ inhibition

System Xc^−^ is essential for the conversion of nutrients into small molecules during ferroptosis. Thus, its transport function is essential for cancer cell survival and growth, making it a potential target for the development of anticancer drugs (Qi et al., 2022). Suppression of system Xc^−^ through erastin or sulfasalazine disrupts cystine uptake, leading to reduced intracellular GSH levels (Li F. J. et al., 2022; Dixon et al., 2014). This reduction impairs protein folding and induces endoplasmic reticulum stress, which is characterized by the upregulation of CHAC1 expression, an enzyme that degrades GSH, to exacerbate oxidative damage and promote ferroptosis (Chen et al., 2017). The compensatory upregulation of SLC7A11 expression was observed following its inhibition, suggesting a feedback mechanism that may serve as a pharmacodynamic biomarker for system Xc^−^ inhibition and ferroptosis (Zheng et al., 2023). These observations suggest that molecular changes associated with system Xc^−^ inhibition may not only reflect target engagement, but may also provide a basis for the development of candidate pharmacodynamic biomarkers.

##### 2.5.1.2 GPX4 inhibition

Although certain cancer cell lines may avoid system Xc^−^ and produce cysteine from methionine through transsulfuration, the inhibition of cystine uptake through systemic Xc^−^ is sufficient to cause ferroptosis. Therefore, system Xc^−^ inhibitors cannot induce ferroptosis in these cells. Alternative methods have consequently been established for ferroptosis induction. For example, GPX4 is a central regulator of ferroptosis. Its inactivation leads to the accumulation of lipid peroxides, which results in ferroptotic cell death (Fu et al., 2019). GPX4 utilizes reduced GSH as a cofactor to catalyze the reduction of lipid hydroperoxides to their corresponding lipid alcohols, thereby limiting the accumulation of lipid ROS and preventing ferroptosis. GPX4 inhibition can induce ferroptosis even at normal GSH and cysteine levels, highlighting its pivotal role in ferroptotic regulation. This underscores the importance of GPX4 in maintaining cellular redox homeostasis and preventing ferroptotic cell death (Yang and Stockwell, 2016; Yang et al., 2016). These findings further support GPX4 as both a therapeutic target and a mechanistically informative candidate marker of ferroptosis vulnerability.

GPX4-specific covalent inhibitors with enhanced tolerance profiles and decreased toxicity are also under development.

##### 2.5.1.3 Nanoparticle inducers

Owing to the unique physicochemical properties of nanomaterials, nanotechnology has recently garnered considerable interest for cancer treatment (Salvioni et al., 2019). The targeting effectiveness, treatment outcomes, and clinical transformation of these nanoplatforms remain inadequate despite increased research on nanotechnology-based anti-cancer techniques (Yu et al., 2023). The rapid development of nano-ferroptosis inducers is an example of a mechanism of ferroptosis that has been considered for nanomedicine-mediated cancer treatment in recent years. Compared with conventional small-molecule inducers, nanoparticle-based systems may improve drug delivery efficiency, enhance tumor or tissue targeting, and increase local iron availability or oxidative stress, thereby promoting lipid peroxidation and ferroptotic cell death. In addition, nanoplatforms can be designed to co-deliver ferroptosis-inducing agents together with chemotherapeutic drugs or other modulators, offering potential advantages for combination therapy. These properties make nanoparticle-based ferroptosis inducers a promising strategy for improving therapeutic efficacy and modulating ferroptosis-related responses. However, their clinical translation remains limited by insufficient targeting specificity, incomplete biosafety evaluation, potential off-target effects, and the lack of robust *in vivo* and clinical validation. Further studies are needed to optimize nanoplatform design and to clarify their translational value in liver diseases and related malignancies.

#### 2.5.2 Therapeutic inhibition of ferroptosis

Iron chelation, prevention of lipid peroxidation, and lipid peroxide scavenging are the three main methods used to inhibit ferroptosis. Iron chelators such as deferoxamine, deferiprone, and ciclopirox bind free iron to limit the Fenton reaction and subsequent lipid peroxidation (Zhang et al., 2025). Lipophilic antioxidants such as Fer-1 and liproxstatin-1 (Lip-1) inhibit ferroptosis by trapping lipid peroxyl radicals, thereby preventing lipid peroxidation (Scarpellini et al., 2023). Despite their efficacy in preclinical models, the clinical applications of Fer-1 and Lip-1 are hindered by several limitations. Fer-1 has a biological half-life of only a few minutes, which is typically unsuitable for use in clinical settings (Li et al., 2025). Although more potent, Lip-1 has demonstrated inhibitory effects on cytochrome P450 2D6, suggesting potential drug–drug interactions that could complicate its clinical application (Sun et al., 2023). In addition to these inhibitors, several other compounds with the potential to inhibit ferroptosis have been identified. These include glutaminolysis inhibitors, beta-mercaptoethanol, dopamine, cycloheximide, and selenium (Chen, 2022). However, no clinical studies have assessed their efficacy in the treatment of liver diseases to date.

Given the promising results of preclinical studies, future research should focus on the development of stronger ferroptosis inhibitors with enhanced pharmacokinetic and pharmacodynamic profiles. These advancements may facilitate the translation of ferroptosis inhibition therapies into clinical settings, thereby offering new avenues for the treatment of liver diseases characterized by excessive ferroptosis.

#### 2.5.3 Ferroptosis and immune modulation in tumors

Beyond direct effects on cell survival, ferroptosis may also influence antitumor immunity through the release of damage-associated molecular patterns and lipid mediators. However, several immune-related mechanisms discussed in this section are based on broader cancer models or non-liver malignancies and have not yet been fully validated in liver disease-specific settings. A small percentage of cells undergoing ferroptosis in the TME can suppress the immune system. This immunosuppression is regulated by DAMPs such as KRAS-G12D, high mobility group box 1 (HMGB1), and 8-hydroxyguanosine (8-OHG) via ferroptosis.

Pancreatic cancer cells release KRAS-G12D, a mutant oncogene, into the extracellular space during ferroptosis. This release is primarily mediated by the formation of amphisomes via fusion of multivesicular autophagosome bodies (Dai et al., 2020). Extracellular KRAS-G12D binds to advanced glycosylation end-product-specific receptor (AGER) on macrophages, leading to their polarization from the M1 (pro-inflammatory) to the M2 (immunosuppressive) phenotype. This shift promotes adaptive immune suppression by increasing the release of immunosuppressive factors such as arginine, IL-10, and TGF-β, thereby stimulating tumor growth (Dai et al., 2020). HMGB1 is another DAMP released during ferroptosis. The released HMGB1 binds to AGER on macrophages, subsequently initiating inflammatory responses through activation of NF-κB signaling (Wen et al., 2019). This interaction contributes to the inflammatory milieu within the TME and supports tumor progression.

8-OHG, a product of oxidative DNA damage, is released during ferroptosis and triggers the macrophage DNA sensing pathway, which is dependent on the stimulation of interferon genes (STING). Pro-inflammatory cytokines such as IL-6 are produced as a result of this activation, thereby creating an inflammatory microenvironment conducive to tumor growth (Liu J. et al., 2021). Ferroptosis is associated with elevated prostaglandin-endoperoxide synthase 2 expression and PGE2 release. PGE2 acts on myeloid-derived suppressor cells and M2 macrophages to inhibit the antitumor functions of NK, dendritic, and cytotoxic T cells. It activates DNA methyltransferase 3A in myeloid cells, leading to DNA methylation and suppression of immunogenic gene expression (Dou et al., 2022). The release of DAMPs during ferroptosis significantly modulates the immune landscape within the TME. Some DAMPs initiate antitumor immune responses, whereas others can promote immune suppression, thereby facilitating tumor progression. Understanding the dual role of ferroptosis in immune modulation is crucial for developing therapeutic strategies that can effectively target ferroptotic pathways to enhance antitumor immunity.

In addition to oxidative stress, ROS regulate immunity in human malignancies by stimulating ferroptosis. Low ROS levels enable T cells to resume their cytotoxic effects. However, excessive ROS production suppresses the production of TCR and MHC antigen complexes in T cells, thereby limiting immunological responses (Su et al., 2023). Additionally, ROS scavengers may enhance CTL activation by activating superoxide dismutase 2. Furthermore, reduced intracellular oxidative stress is associated with the ability of CAR-T cells to eradicate cancer cells. Therefore, CAR-T cells combined with catalase (an ROS inhibitor) demonstrated an enhanced antitumor response even when the extracellular surface was subjected to oxidative stress.

Although much of the current evidence derives from tumor models rather than liver disease-specific settings, these findings suggest that ferroptosis may also influence immune microenvironmental responses during disease progression and therapy. Therefore, these findings should be interpreted as hypothesis-generating rather than definitive evidence for liver diseases. Further studies are needed to determine whether ferroptosis-associated immune mechanisms are conserved in HCC, chronic hepatitis, fibrosis-associated inflammation, and NAFLD/MASLD-related immune remodeling, and whether such mechanisms can support clinically useful biomarkers or therapeutic strategies.

#### 2.5.4 Limitations in the clinical validation of ferroptosis-related biomarkers

Although ferroptosis-related molecules show considerable translational promise, their clinical validation as biomarkers in liver diseases remains limited (Zhou et al., 2024; Peleman et al., 2024b). First, many studies are based on small cohorts, single-center datasets, or preclinical models, which limits generalizability. Second, independent validation across external cohorts is still insufficient for most candidate markers. Third, assay platforms vary substantially, including immunohistochemistry, transcriptomics, proteomics, metabolomics, and biochemical lipid peroxidation assays, making cross-study comparisons difficult. Fourth, ferroptosis is highly tissue-, cell-type-, and context-dependent; therefore, signals detected in serum or bulk tissue may not accurately reflect ferroptotic activity in specific cell populations, such as hepatocytes, hepatic stellate cells, immune cells, or tumor cells. Finally, prospective studies are needed to determine whether ferroptosis-related biomarkers can improve risk prediction, patient stratification, treatment selection, or treatment-response monitoring beyond established clinical and pathological parameters. These limitations should be addressed before ferroptosis-related biomarkers can be implemented in routine clinical practice.

## 3 Conclusion and future prospects

Ferroptosis is involved in the onset, progression, and therapeutic modulation of multiple liver diseases. Therefore, we reviewed its underlying mechanisms and highlighted its role in several liver disorders. Current evidence indicates that ferroptosis contributes not only to HCC progression, but also to hepatocyte injury and fibrogenesis in NAFLD/MASLD, ALD, acute liver injury, and liver fibrosis.

With a better understanding of ferroptosis, key regulators can be modulated to prevent or trigger this process, thereby slowing the course of liver disease and facilitating the development of novel therapeutic strategies. In particular, the prevention of liver damage caused by lipid peroxidation, inflammatory infiltration, and immunogenicity may benefit from the targeted regulation of ferroptosis. At the same time, the context-dependent role of ferroptosis suggests that it may serve not only as a therapeutic target, but also as a source of candidate biomarkers for disease evaluation and therapeutic stratification.

The discovery of ferroptosis has created new avenues for liver disease research, and its importance in disease onset, progression, and therapeutic modulation has become increasingly apparent. However, ferroptosis research in liver diseases remains at an early translational stage. Future studies should further clarify context-specific mechanisms, standardize biomarker detection methods, validate ferroptosis-related markers in disease-specific and prospective clinical cohorts, and develop tailored therapeutic strategies. These efforts will be essential for translating ferroptosis biology into clinically useful biomarkers and precision medicine approaches for liver diseases.

## Glossary

AA: arachidonic acid
AIH: autoimmune hepatitis
ALD: alcohol-associated liver disease
ALI: acute liver injury
APAP: acetaminophen
ATF4: activating transcription factor 4
BH4: tetrahydrobiopterin
CLD: chronic liver disease
CoQ10: coenzyme Q10
DAMPs: damage-associated molecular patterns
DHODH: dihydroorotate dehydrogenase
DILI: drug-induced liver injury
DMT1: divalent metal transporter 1
DPP4: dipeptidyl-peptidase-4
Fer-1: ferrostatin-1
FPN: ferroportin
FSP1: ferroptosis suppressor protein 1
GCH1: GTP cyclohydrolase-1
GPX4: glutathione peroxidase 4
GSH: glutathione
HBV: hepatitis B virus
HCC: hepatocellular carcinoma
HCV: hepatitis C virus
HFD: high-fat diet
HMGB1: high mobility group box 1
HSCs: hepatic stellate cells
IFN-γ: interferon-gamma
IRI: ischemia-reperfusion injury
KEAP1: Kelch-like ECH-associated protein 1
LIP: labile iron pool
Lip-1: liproxstatin-1
LOX: lipoxygenase
MASLD: metabolic dysfunction-associated steatotic liver disease
MASH: metabolic dysfunction-associated steatohepatitis
MDA: malondialdehyde
NAFLD: nonalcoholic fatty liver disease
NASH: nonalcoholic steatohepatitis
Nrf2: nuclear factor erythroid 2-related factor 2
NTBI: non-transferrin-bound iron
PGE2: prostaglandin E2
PUFAs: polyunsaturated fatty acids
ROS: reactive oxygen species
SLC7A11: solute carrier family 7 member 11
STEAP3: six-transmembrane epithelial antigen of prostate 3
Tf: transferrin
TfR1: transferrin receptor 1
TME: tumor microenvironment
xCT: cystine/glutamate antiporter
